# Compositions and Abundances of Sulfate-Reducing and Sulfur-Oxidizing Microorganisms in Water-Flooded Petroleum Reservoirs with Different Temperatures in China

**DOI:** 10.3389/fmicb.2017.00143

**Published:** 2017-02-02

**Authors:** Huimei Tian, Peike Gao, Zhaohui Chen, Yanshu Li, Yan Li, Yansen Wang, Jiefang Zhou, Guoqiang Li, Ting Ma

**Affiliations:** Key Laboratory of Molecular Microbiology and Technology, Ministry of Education, College of Life Sciences, Nankai UniversityTianjin, China

**Keywords:** sulfate-reducing bacteria (SRB), corrosion, sulfur-oxidizing bacteria (SOB), oil reservoirs, diversity, abundance

## Abstract

Sulfate-reducing bacteria (SRB) have been studied extensively in the petroleum industry due to their role in corrosion, but very little is known about sulfur-oxidizing bacteria (SOB), which drive the oxidization of sulfur-compounds produced by the activity of SRB in petroleum reservoirs. Here, we surveyed the community structure, diversity and abundance of SRB and SOB simultaneously based on 16S rRNA, *dsrB* and *soxB* gene sequencing, and quantitative PCR analyses, respectively in petroleum reservoirs with different physicochemical properties. Similar to SRB, SOB were found widely inhabiting the analyzed reservoirs with high diversity and different structures. The dominant SRB belonged to the classes *Deltaproteobacteria* and *Clostridia*, and included the *Desulfotignum*, *Desulfotomaculum*, *Desulfovibrio*, *Desulfobulbus*, and *Desulfomicrobium* genera. The most frequently detected potential SOB were *Sulfurimonas*, *Thiobacillus*, *Thioclava*, *Thiohalomonas* and *Dechloromonas*, and belonged to *Betaproteobacteria*, *Alphaproteobacteria*, and *Epsilonproteobacteria*. Among them, *Desulfovibrio*, *Desulfomicrobium*, *Thioclava*, and *Sulfurimonas* were highly abundant in the low-temperature reservoirs, while *Desulfotomaculum*, *Desulfotignum, Thiobacillus*, and *Dechloromonas* were more often present in high-temperature reservoirs. The relative abundances of SRB and SOB varied and were present at higher proportions in the relatively high-temperature reservoirs. Canonical correspondence analysis also revealed that the SRB and SOB communities in reservoirs displayed high niche specificity and were closely related to reservoir temperature, pH of the formation brine, and sulfate concentration. In conclusion, this study extends our knowledge about the distribution of SRB and SOB communities in petroleum reservoirs.

## Introduction

Oil reservoirs represent anaerobic environments and harbor diverse microbial populations, including hydrocarbon-oxidizing, fermentative, sulfur-oxidizing, sulfate-reducing, and methanogenic bacteria (Voordouw et al., 1996; Ollivier et al., 1997; Magot et al., 2000; Dong et al., 2014). These microorganisms play important roles in the biogeochemical cycles of carbon, nitrogen, and sulfur in reservoir ecosystems. As sulfate-reducing bacteria (SRB) are commonly considered the main culprits of corrosion which causes great economic loss in oil industry, they have gained prominence (Gieg et al., 2011; Guan et al., 2013; Drønen et al., 2014). Based on the study of Enning and Garrelfs, SRB can induce the corrosion of iron in two different ways: indirectly produce the corrosive chemical agent hydrogen sulfide (H_2_S) [chemical microbially influenced corrosion (CMIC)] and directly attack iron via withdrawal of electrons [electrical microbially influenced corrosion (EMIC)] (Enning and Garrelfs, 2014). Serious corrosion phenomena are also present in the marine industry and gas industry pipelines where SRB were commonly detected and were the main players (Zhu et al., 2003; Païssé et al., 2013; Stipanicev et al., 2014). Also, sewer systems have been found to suffer concrete corrosion and it was primarily caused by SOB which oxidize the dissolved H_2_S and other sulfur compounds to sulfuric acid (Okabe et al., 2007).

Realizing the severity of the corrosion, many protective measures against SRB are widely studied (Hubert and Voordouw, 2007; Bodtker et al., 2008; Voordouw et al., 2011). Currently, nitrate injection is considered the most effective way which largely stimulated NRB, an inhibitor for SRB, and they have been extensively investigated in large-scale studies (Bodtker et al., 2008; Voordouw et al., 2011). NRB compete with SRB for organic electron donors and produce inhibitory concentrations of nitrite that inhibit the growth of SRB in oil fields (Hubert and Voordouw, 2007). Similar to NRB, nitrate-reducing sulfide-oxidizing bacteria (NR-SOB) may compete with SRB for degradable oil constituents. In addition, many SOB have also been reported to decrease the concentration of sulfide, which is toxic to human and inhibits the production of rhamnolipids (Mattorano and Merinar, 1999; Gusseme et al., 2009; Zhao et al., 2015). However, *Epsilonproteobacteria* of the genera *Sulfuricurvum* and *Sulfurovum* belonging to SOB play a potential role in microbiologically influenced corrosion (MIC) in pipelines subjected to injection of bisulfite for the scavenging of oxygen (An et al., 2016). Catalyzing the opposite reaction of sulfate reduction, SOB have gain little attention in its distribution in oil reservoirs.

The biological sulfur cycle, which includes sulfate reduction and sulfur oxidation, is an integral part of the biogeochemical cycles in reservoirs. It connected to many other main element cycles, such as carbon (oxidation of organic compounds, formation of organo-sulfur compounds), nitrogen (thiodenitrification), and metal (formation of metal sulfides) cycles. Sulfate reduction is driven by strict anaerobic SRB that can obtain energy by oxidizing organic compounds or molecular hydrogen (H_2_) while reducing sulfate (SO_4_^2-^) to hydrogen sulfide (H_2_S) (Muyzer and Stams, 2008). The SRB found in oil reservoirs primarily belong to *Deltaproteobacteria*, *Firmicutes*, *Nitrospira*, *Thermodesulfobacterium*, and *Euryarchaeota* (Youssef et al., 2009; Gieg et al., 2011). Sulfur oxidation refers to the oxidization of inorganic sulfur compounds into sulfate and this process is catalyzed by SOB. SOB communities constitute physiologically and phylogenetically diverse members of *Alpha*-, *Beta*-, *Gamma*- and *Epsilonproteobacteria*, *Chlorobia*, and *Chloroflexi* (Ghosh and Dam, 2009). *Paracoccus*, *Thiobacillus*, *Sulfurospirillum*, *Sulfuricurvum*, *Sulfurovum*, and *Sulfurimonas* have been found in petroleum reservoirs (Gieg et al., 2011; Silva et al., 2013; Gao et al., 2015a; An et al., 2016). These microorganisms drive sulfur oxidation via three different pathways: the *Paracoccus* sulfur oxidation (PSO), branched thiosulfate oxidation, and tetrathionate intermediate (S_4_I) pathways (Friedrich et al., 2005; Hensen et al., 2006; Ghosh and Dam, 2009; Gregersen et al., 2011).

Although SOB are closely related to SRB and play a potentially role in MIC, the distribution of SOB in reservoir environments have rarely been studied. Here, we investigated the diversity and composition of SRB and SOB communities in three subterranean oil reservoirs with diverse physicochemical properties to know how they distributed and which factor dominated their distribution in each reservoir. Due to their high phylogenic diversity, SRB and SOB populations were determined using 16S rRNA gene high-throughput sequencing coupled with clone libraries of the functional genes *dsrB* and *soxB*. The *dsrB* gene encodes major subunits of dissimilatory (bi) sulfite reductase (DsrB), which is required for the reduction of sufite to sulfide, and has been used as a phylogenetic marker for the identification of SRB (Geets et al., 2006; Giloteaux et al., 2013; Guan et al., 2013; Gao et al., 2015b). The *soxB* gene has been detected in sulfur oxidizers irrespective of the pathway and thus it was selected as the biomarker of SOB in our study (Friedrich et al., 2005; Meyer et al., 2007). The combination of 16S rRNA gene and functional gene sequencing improves our ability to analyze these microbial populations in detail. In addition, quantitative PCR was used to reveal the abundance of SRB and SOB populations in these reservoirs.

## Experimental Procedures

### Site Description and Sampling

Water samples were collected from three water-flooded petroleum reservoirs in China. The LZ and QZ petroleum blocks are located at the L-field block and Q-field block reservoir areas of the Karamay oil field, Xinjiang Oil Field, Co. Ltd, PetroChina. The DQ reservoir is located at the N2-field block reservoir area of Daqing oil field, China, and the SL reservoir is located in the Yellow River Delta of China, Dongying, near the Bohai Sea. The three reservoirs have been flooded for over 30 years with different geochemical conditions. For the LZ block of Xinjiang reservoir, the average depth of the petroleum horizons is 480 m, with a formation temperature of 22°C and an average permeability of 0.362 μm^2^. The viscous oil recovered from the production wells in this block has a viscosity of approximately 80 mPa⋅s. On the other hand, the QZ block has a formation temperature of 37°C, average depth of 1088 m, and an average permeability of 0.123 μm^2^ and the viscosity of crude oil is 5.6 mPa⋅s. The depth of the DQ reservoir is approximately 1054 m underground, with an average temperature of 46°C. The average permeability is 1.32 μm^2^ and the viscosity of crude oil is 9.2 mPa⋅s. The SL reservoir is a high-temperature reservoir of about 66°C with an average depth of 1150 m. The average permeability is 1.673 μm^2^ and the viscosity of viscous oil recovered from the production wells in this block is 46 mPa⋅s.

Production oil/water mixture samples were collected directly from each wellhead of the production well by field personnel of PetroChina. Each sample was collected in triplicate. 10 L of sterilized plastic bottles were completely filled by samples and they were immediately sealed with screw caps to avoid contamination and oxygen intrusion. The bottles were then transported to the laboratory as soon as possible for further analysis. After separating the oil and water phases, 5 L of each water sample was centrifuged at 4°C for 15 min at 10,000 *g* in a high-speed centrifuge (Beckman, Pasadena, CA, USA) to collect microbial cells within 24 h of collection. Then, 100 mL of cell-free water samples were stored at 4°C for chemical analysis. Chemical analyses conducted on the samples included the measurement of pH, concentrations of cations and anions. Ion chromatograph (DIONEX ICS-1000) was used to analyze the concentrations of cations (with a Shim-pack IC-A3 column) and anions (with a Shim-pack IC-C3 column).

### Genome Extraction and High-Throughput Sequencing of Partial 16S rRNA Genes

Total genomic DNA was extracted from the cell residue using methods previously described (Zhao et al., 2012). The collected cells were resuspended in TE buffer (80 mM Tris, 40 mM EDTA; pH 8.0). Then, 0.1 mm glass beads were added to lysis cells using a mini beadbeater (BioSpec, Bartlesville, OK, USA) at 200 rpm for 1 min at room temperature. DNA was extracted from the suspension using AxyPrep Bacterial Genomic DNA Miniprep Kit (Axygen Biosciences, Union City, CA, USA) according to the manufacturer’s instructions. After extraction, the DNA quantity and concentration were measured by 1% agarose gel electrophoresis and Biodrop μLite Spectrophotometer (BioDrop, Cambridge, UK), respectively.

Pyrosequencing was performed by Majorbio Company (Shanghai, China) using a Roche 454 FLX + platform (Roche) for LZ and SL samples. Bacterial 16S rRNA gene sequences were amplified using the universal primers 27F (AGAGTTTGATCCTGGCTCAG) and 533R (TTACCGCGGCTGCTGGCAC). For sequencing, the A linker and unique Roche multiplex identifiers (MID) were added to the 533R primer. The barcode was permuted for each sample to identify the individual samples in a mixture in a single pyrosequencing run. Each sample was amplified in a 20-μL PCR reaction mixture with 0.25 μM of each primer for three replicates. PCR was conducted with initial denaturation at 95°C for 2 min followed by 30 cycles at 94°C for 30 s, 50°C for 30 s, and 72°C for 30 s, and a final elongation step at 72°C for 5 min. Amplicons from different water samples were then mixed to achieve equimolar concentrations in the final mixture for sequencing.

MiSeq-sequencing was adopted for QZ and DQ samples. Bacterial 16S rRNA gene V4 region (300–350 bp) were amplified using primer set 515f (GTGCCAGCMGCCGCGGTAA) and 806r (GGACTACHVGGGTWTCTAAT) with the protocol described by Caporaso et al. (2012). A composite sample for sequencing was created by combining equimolar ratios of amplicons from the individual samples, followed by gel purification to remove any remaining contaminants and PCR artifacts. Amplicon sequencing was conducted on an Illumina MiSeq platform at Majorbio, Co., Shanghai, China.

### Clone Libraries Construction of the *dsrB* and *soxB* Genes

The *dsrB* and *soxB* genes were amplified using the extracted DNA. The reactions for the two genes were performed in a 50-μL volume containing 25.0 μL of Ex Taq Premix (TaKaRa Biotechnology, Co., Ltd, Dalian, China), 2.0 μL of genomic DNA, and 0.4 μM of the specific primers for each gene. Sets of DSRp2060F (CAACATCGTYCAYACCCAGGG) and DSR4R (GTGTAGCAGTTACCGCA) were used to amplify the *dsrB* gene in a reaction with 30 cycles of 95°C for 45 s, 55°C for 1 min and 72°C for 1 min (Wagner et al., 1998; Geets et al., 2006; Varon-Lopez et al., 2013). Similarly, *soxB* gene was amplified with 710F (ATCGGYCAGGCYTTYCCSTA) and 1184R (MAVGTGCCGTTGAARTTGC) in a reaction with cycles of 95°C for 30 s, 55°C for 30 and 72°C for 40 s (Tourna et al., 2014). After the samples were electrophoresed in 1.0% agarose gel, products of the desired target size (380 and 511 bp for the *dsrB* and *soxB* genes, respectively) were excised from the gels and purified with the Axygen miniprep DNA purification kit (Axygen Biosciences, Union City, CA, USA). The purified PCR products were directly ligated into the pMD19-T Simple vector (TaKaRa Biotechnology, Co., Ltd) and transformed into competent *Escherichia coli* cells (TransGen Biotechnology, Co., Ltd, Beijing, China). The correct inserts were screened by PCR using the vector-special primer set RV-M (GAGCGGATAACAATTTCACACAGG) and M13-47 (CGCCAGGGTTTTCCCAGTCACGAC). Amplified ribosomal DNA restriction analysis (ARDRA) was used to classify the PCR products of positive clones with *HhaI* and *HaeIII* (TaKaRa Biotechnology, Co., Ltd) (Zhang et al., 2010). Clones with identical ARDRA profiles were classified into one operational taxonomic unit (OTU). Representative clones from different OTUs were selected for sequencing by an automated ABI 3730 DNA sequencer.

### Sequence Analysis of the 16S rRNA, *dsrB* and *soxB* Genes

The raw data of LZ and SL samples were processed and analyzed by the open-source software Mothur^1^ (Schloss et al., 2009). All sequences were sorted into different samples according to barcodes. Sequences were subjected to systematic checks to remove replicates, duplicates, barcodes, primer sequences and low-quality reads. Briefly, high quality sequences (>200 bp in length, quality score >25, exact match to barcode and primer, and containing no ambiguous characters) were remaining. The remaining sequences were grouped into OTUs by setting a 0.03 distance limit (equivalent to 97% similarity). Coverage and alpha diversity analyses including computation of the Shannon, Simpson and Chao 1 indexes were used to assess biodiversity based on OTUs.

For QZ and DQ samples, pairs of reads from the original DNA fragments were merged using FLASH (fast length adjustment of short reads) (Magoć and Salzberg, 2011). Raw sequences were demultiplexed and quality-filtered using the default parameters in QIIME software package (Quantitative Insights Into Microbial Ecology) to obtain the high-quality clean tags (Caporaso et al., 2010; Bokulich et al., 2013). Chimera sequences were detected by UCHIME algorithm and were removed (Edgar, 2011). Then, sequences were classified to OTUs at 97% similarity using Uparse pipeline and representative sequence for each OTU was screened (Edgar, 2013). Alpha diversity analyses including computation of the Shannon, Simpson, Chao 1 indexes and Coverage were used to assess community richness, diversity and sequencing depth based on OTUs.

The nucleotide sequences of the *dsrB* and *soxB* genes were processed to remove primers and vector sequences using VecScreen^2^, and the chimeras were excluded using Chimera Check program^3^. Poor-quality sequences were also excluded from the analysis. The DNA sequences were analyzed using BLASTn to identify their phylogenetic affiliations and to remove non-specific sequences. The processed sequences and its closely related sequences obtained from GenBank^4^ were aligned by Clustal X 1.81. Bacteria diversity was assessed using the Shannon-Wiener diversity index (H), Simpson diversity index (D), and Pielou evenness index (E). Individual clone library coverage was also calculated. Phylogenetic trees based on the *dsrB* and *soxB* genes were constructed by the neighbor-joining method with 500 bootstrap replicates using MEGA6 (Tamura et al., 2013).

### Effect of Environmental Factors on Community Distributions

Abundance data from the partial 16S rRNA gene sequencing and functional genes clone libraries were normalized for each sample. These data were then used to evaluate the relationship between the major functional microorganism groups and environmental factors such as temperature, pH, and mineralization by stepwise canonical correspondence analysis (CCA) using Canoco software.

### Quantification of *dsrB*, *soxB*, and 16S rRNA Genes

The copy numbers of *dsrB*, *soxB*, and 16S rRNA genes were determined by qPCR. Reactions were performed using the Bester SybrGreen qPCR Mastermix (DBI Bioscience, Germany) in a Bio-Rad iQ5 Sequence detection system (Applied Biosystems, Carlsbad, CA, USA). The *dsrB* and *soxB* genes were amplified with the same primers and PCR reaction conditions used for clone libraries. The 16S rRNA gene was amplified using the following primer sets: 8F (AGAGTTTGATYMTGGCTC) and 338R (GCTGCCTCCCGTAGGAGT) (Gittel et al., 2009). The cycle parameters were as follows: initial denaturation at 95°C for 3 min followed by 40 cycles of 94°C for 30 s, 55°C for 30 s, and 72°C for 30 s. Standard curves were constructed by using 10-fold serial dilutions of standard plasmids with a known number of copies of template DNA to quantify the targeted genes. Plasmid standards and environmental samples were simultaneously assayed in triplicate. The specificity of amplification was verified using a denaturation curve from 55 to 98°C at the end of each reaction. Standard curves with efficiencies from 90 to 110% and corresponding *R*-value of >0.99 were considered credible results.

### GenBank Submission and Accession Numbers

All the validated nucleotide sequence data obtained from clone libraries in this study were deposited in the GenBank database with the accession numbers KU603312–KU603333 and KU603334–KU603352 for the *dsrB* and *soxB* genes, respectively. The sequencing reads were deposited in the National Center for Biotechnology Information (BioProject ID: PRJNA356912).

## Results

### Geochemical Characteristics of the Petroleum Reservoirs

Water samples were collected from eight production wells located in three geographically isolated reservoirs. The distances between each of the reservoirs ranged from 10s to 1000s of kilometers. Although the LZ and QZ reservoirs are in the same oilfield, they have different underground oil-bearing strata. **Table 1** summarizes the geochemical features of the target reservoirs. The most obvious difference between the reservoirs was the temperature, which ranged from 22 to 66°C. Another clear difference was the pH of the formation brines, which ranged from 5.8 to 7.8. Furthermore, the samples from different reservoirs were characterized by various SO_4_^2-^ concentrations ranging from 32.7 to 245.6 mg/L, indicating that sulfate reduction could occur. The mineralization of the water samples varied from 4530.6 to 12193.1 mg/L and the Cl^-^ concentration ranged from 722.5 to 5329.6 mg/L. The concentrations of divalent ions such as Mg^2+^ and Ca^2+^ and monovalent ions such as Na^+^ and K^+^ varied from 43.4–144.2 mg/L and 1373.2–4069.9 mg/L, respectively.

**Table 1:** Geochemical characteristics of production water samples from eight wells in target reservoirs.

Reservoir samples	Wells	Temp (°C)	pH	Mineralization^#^ (mg/L)	SO_4_^2-^ (mg/L)	Water type	Na^+^ +K^+^ (mg/L)	Mg^2+^/Ca^2+^ (mg/L)	Cl^-^ (mg/L)
LZ	LZ89	22	7.6	12193.1	87.4	NaHCO_3_	4069.9	99.6	4356.2
	LZ96	22	7.7	11177.7	47.8	NaHCO_3_	3659.5	144.2	3916.2
QZ	QZ53	34	7.7	11387.1	245.6	Na_2_SO_4_	3817.9	135.3	4387.8
	QZ02	34	7.8	11618.4	78.3	Na_2_SO_4_	3853.2	143.7	4387.8
DQ	DQ41	45	6.4	11900.2	85.5	NaHCO_3_	3430.7	59.5	1220.3
	DQD2	45	6.7	4530.6	32.7	NaHCO_3_	1330.8	43.4	722.5
SL	SL21	66	5.8	9642.0	52.3	NaHCO_3_	1677.2	98.7	5329.6
	SL22	66	5.8	6778.0	64.5	NaHCO_3_	1373.2	89.5	3520.8

*#Mineralization represents the concentration of total irons in water samples*.

### SRB and SOB Inferred from 16S rRNA Gene High-Throughput Sequencing

For high-throughput sequencing, after filtering the low-quality reads and chimeras, 159, 813, 781, and 522 OTUs based on the 97% cutoff of effective 16S rRNA sequences were collected from the LZ, QZ, DQ, and SL reservoirs, respectively. **Table 2** summarizes the number of reads, OTUs and the Chao 1, ACE, Shannon, and Simpson indices. The coverage of the sequencing libraries reached 98%, which could reflect the entire microbial community in each production water sample. Based on general species classification, a total of 23 genera of potential SRB were inferred from the total bacterial community accounting for 0.98, 0.57, 0.77, and 13.57% of total bacteria in LZ, QZ, DQ and SL reservoirs, respectively. Among the detected SRB genera, *Desulfovibrio*, *Desulfotignum*, *Desulfotomaculum*, *Desulfobulbus*, *Desulfomicrobium*, and *Desulfurivibrio* were dominated, while *Desulfocapsa*, *Desulfurivibrio*, *Desulfosarcina*, *Desulfococcus*, *Desulfomonile* were rare in these reservoirs (Supplementary Table S1). The 16S rRNA sequencing results have identified 21 genera as potential SOB and they were mainly belonged to *Alphaproteobacteria*, *Betaproteobacteria*, *Gammaproteobacteria*, and *Epsilonproteobacteria*. Those potential SOB occupied 0.45, 6.54, 0.93, and 14.96% for LZ, QZ, DQ, and SL reservoirs, respectively. Among them, *Thioclava*, *Roseovarius*, *Sulphurospirillum*, and *Thiohalomonas* were dominated while *Rhizobium*, *Thiobacillus*, *Sulfuricurvum*, *Hydrogenophaga*, *Thiohalomonas*, *Paracoccus*, *Thioalkalispira*, *Thiofaba*, *Thiothrix*, and *Thiovirga* were detected with low abundances (Supplementary Table S2).

**Table 2:** Biodiversity analysis of the 16S rRNA sequences retrieved from high-throughput sequencing of targeted petroleum reservoirs.

Reservoir samples	Samples	n.read	n.OTU	ACE	Chao	Coverage (C)	Shannon (H)	Simpson (D)
LZ	LZ89	2629	88	190	141	0.985	1.82	0.361
	LZ96	3388	71	102	100	0.991	1.63	0.329
QZ	QZ53	37012	410	505	515	0.997	3.69	0.052
	QZ02	53340	403	657	576	0.997	2.49	0.274
DQ	DQ41	38801	569	986	938	0.993	4.92	0.976
	DQD2	38221	212	316	308	0.991	1.63	0.347
SL	SL21	5538	185	251	268	0.989	3.04	0.108
	SL22	6502	337	395	392	0.988	4.39	0.028

### SRB and SOB Detected by *dsrB* and *soxB* Gene Clone Libraries

**FIGURE 1:**
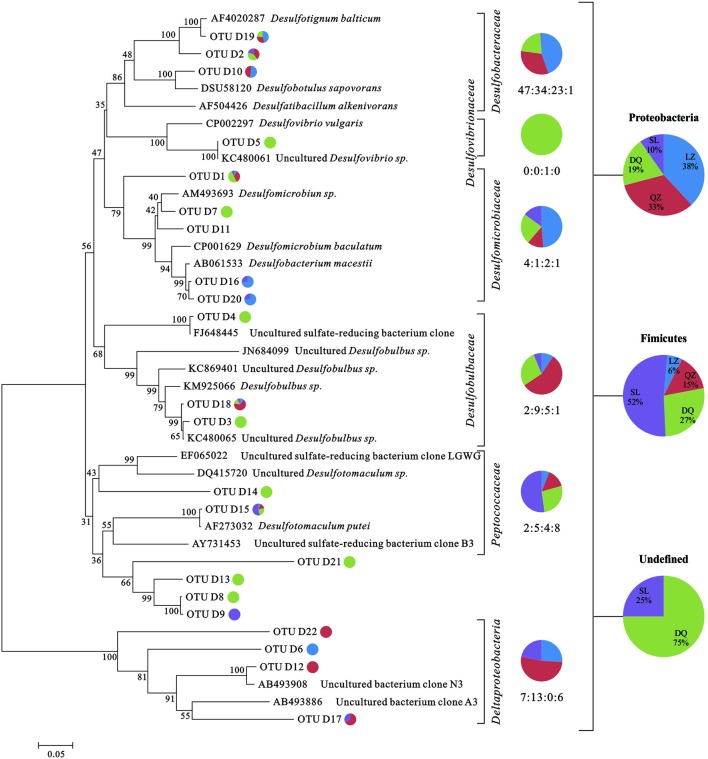
**Phylogenetic trees based on clone libraries of *dsrB* gene from different oil reservoirs**. The pie diagram and the numbers under the diagram display the number of sequences allocated per oil reservoir (blue-LZ block, red-QZ block, green-DQ reservoir, purple-SL reservoir) in each OTU. The phylogenetic analyses were carried out by using one representative sequence from each OTU. Each representative sequence was compared with the public database (GenBank and Fungene), and the sequences with the best matches were used for the alignments and phylogenetic construction.

**FIGURE 2:**
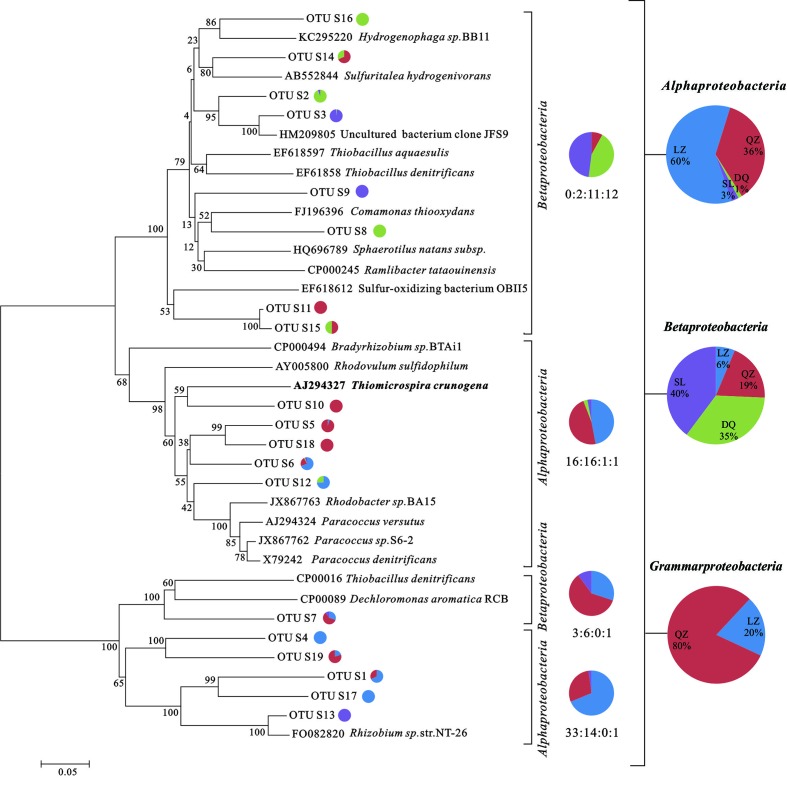
**Phylogenetic trees based on clone libraries of *soxB* genes from different oil reservoirs**. The pie diagram and the numbers under the diagram display the number of sequences allocated per oil reservoir (blue-LZ block, red-QZ block, green-DQ reservoir, purple-SL reservoir) in each OTU. The phylogenetic analyses were carried out by using one representative sequence from each OTU. Each representative sequence was compared with the public database (GenBank and Fungene), and the sequences with the best matches were used for the alignments and phylogenetic construction. The bold marked species was the exception of their senior affiliation listed in the right.

For *dsrB* clone librarirs, 465 sequences were finally obtained and were divided into 126 OTUs. The Shannon-Weiner index (H), Simpson index (D), Dielou evenness index (E) and the coverage (C) of each gene were estimated (Supplementary Table S3). 7 SRB genera, including *Desulfobacterium*, *Desulfotignum*, *Desulfovibrio*, *Desulfomicrobium*, *Desulfotomaculum*, *Desulfobotulus*, were detected with more than 90% of similarity. As shown in the phylogenetic tree of *dsrB* sequences, the majority of genera were associated to *Desulfobacteracceae*, *Desulfovibrionaceae*, *Desulfomicrobiaceae*, *Desulfobulbaceae*, and *Peptococcaceae*. Besides, there were sequences only assigned to *Deltaproteobacteria* class level (**Figure 1**). From the results, members assigned to *Deltaproteobacteria* were more abundant than that assigned to *Clostridia*. For *soxB* gene clone libraries, 454 sequences were finally obtained and they were divided into 109 OTUs (Supplementary Table S3B). Those sequences were affiliated to seven known genera and two uncultured taxa with more than 80% of similarity. *Rhizobium*, *Paracoccus*, *Thiomicrospira*, *Hydrogenophaga*, *Bradyrhizobium*, and *Thiobacillus* were detected. As shown in phylogenetic tree of *soxB* sequences (**Figure 2**), genera were affiliated to *Betaproteobacteria* which contained three orders that were *Burkholderiales*, *Rhodocyclales* and *Hydrogenophilales*, and *Alphaproteobacteria* that included *Rhizobiales* and *Rhodobacteriales*.

### SRB Community in Petroleum Reservoirs

**FIGURE 3:**
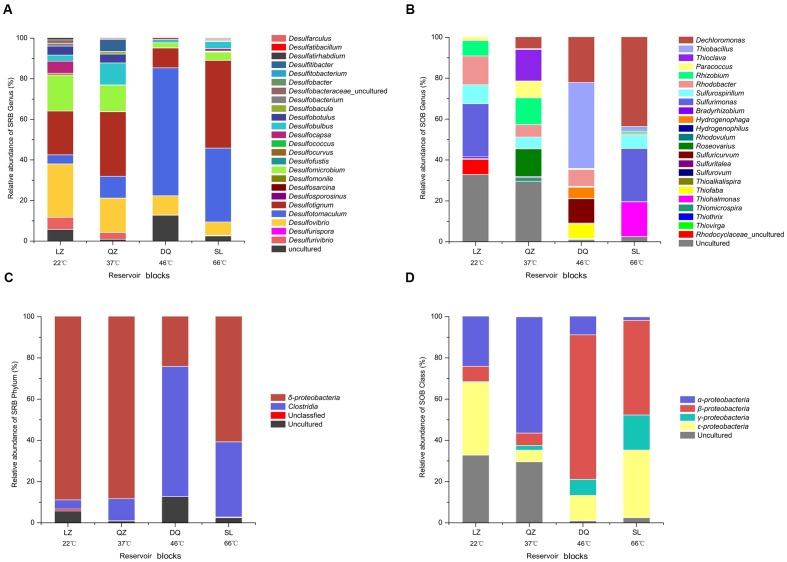
**Assignment of bacteria retrieved from merged results affiliated to SRB and SOB at different levels in targeted reservoirs** (**A**, SRB at genus level; **B**, SOB at genus level; **C**, SRB at class level; **D**, SOB at Class level).

After merging the results from 16S rRNA pyrosequencing and *dsrB* clone librarirs in the percentage level, 24 SRB genera, including *Desulfovibrio*, *Desulfotignum*, *Desulfomicrobium*, *Desulfobulbus*, *Desulfotomaculum*, *Desulfomicrobium*, *Desulfocapsa*, and *Desulfococcus* were detected (**Figure 3A**). The compositions of the SRB community differed among the three reservoirs. *Desulfovibrio*, *Desulfotignum*, and *Desulfomicrobium* were the dominant genera among 13 genera in the LZ block with 26.3, 21.7, and 17.7%, respectively. For QZ block, 16 SRB genera were detected with *Desulfotignum* (31.8%), *Desulfovibrio* (17.0%), and *Desulfomicrobium* (13.0%) as majority. *Desulfotomaculum*, commonly occurs in the deep biosphere and may play an essential role in the dynamics of deeply buried anaerobic bacterial communities (Aüllo et al., 2013), was detected at a higher proportion in the DQ (9 SRB genera) and SL (22 SRB genera) reservoirs (63.1 and 36.4%, respectively), which have relatively high temperatures. Apart from *Desulfotomaculum* genus, *Desulfotignum* occupied about 43.2% for SL reservoirs.

**FIGURE 4:**
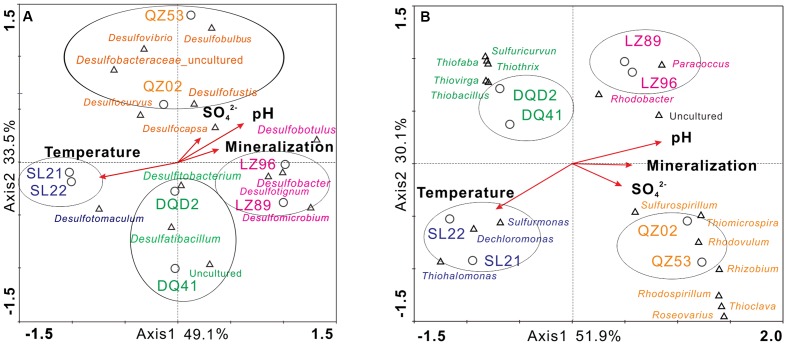
**Canonical correspondence analysis (CCA) of merged results in target reservoirs along with the selected physicochemical characteristics of production water samples**. Genera in different colors represent different affiliations (pink-LZ block, orange-QZ block, green-DQ reservoir, purplish blue-SL reservoir); species marked in black are dominant bacterial species shared by at least two reservoirs (**A**, SRB; **B**, SOB).

To illustrate the relationship between the microbial communities and the environmental variables in petroleum reservoirs, CCA was conducted to analyze the community composition and the major physiochemical parameters, including temperature, sulfate concentration, pH, and mineralization. The results indicated that approximately 80% of community variation could be explained by the two axes of the CCA biplot. As shown in **Figure 4A**, the SRB communities displayed high niche specificity, i.e., microbial communities from the same reservoir block formed clusters that differed from those of other reservoir blocks. In the relatively low temperature reservoirs (LZ and QZ), temperature had little influence on the SRB community composition, while the pH and mineralization of formation brines were the main factors influencing the SRB population in the LZ block, and the concentration of sulfate ions affected the SRB community composition in the QZ block. In the relatively high temperature reservoirs (SL), considerable effects were played on the SRB community by temperature.

### SOB Community in Petroleum Reservoirs

Here, 22 different potential SOB genera, including *Thiohalmonas*, *Sulfurimonas*, *Thioclava*, *Thiobacillus*, *Rhizobium*, *Sulfurovum*, *Sulfurospirillum*, *Thiofaba*, *Rhodobacter*, and *Dechloromonas* were detected based on the merged result (**Figure 3B**). The community structure of the SOB communities varied across the different reservoirs. The *Sulfurimonas* genus belonging to class *Epsilonproteobacteria* was the dominant one in the LZ reservoir (26.0%) and occupied high proportion (26.1%) in the high-temperature SL reservoir (26.1%), indicating that this genus is capable of growth at a wide range of temperatures. As previously reported, many members of *Sulfurimonas* can grow at 10–40°C, but *Thermosulfurimonas* can grow at 50–92°C (Inagaki et al., 2003; Slobodkin et al., 2012). Moreover, *Sulfurimonas* were most frequently detected in surface sediment sample with high temperatures (Gugliandolo et al., 2015). Uncultured bacteria were detected with high proportion in LZ (32.9%) and QZ (29.7%) blocks. For the QZ reservoir, *Thioclava*, which is a typical genus of *Alphaproteobacteria*, represented approximately 15.4%. The *Thiobacillus* genus, belonging to *Betaproteobacteria* that has been reported can be stimulated by the increase in petroleum levels (Peixoto et al., 2011), represented 42.0% of the SOB in the DQ reservoir in this study. Bacteria of this genus derive energy from the oxidation of reduced sulfur compounds (sulfide, elemental sulfur, and thiosulfate) to sulfate. *Sulfuricurvum* (12.0%) and *Thiohalomonas* (17.0%) were another prevailing genus for DQ and SL reservoir, respectively. Possessing *soxB* gene, *Dechloromonas* strain RCB dominated in the SL reservoir, but no study has been found on its function in sulfur oxidation. However, studies have found that this strain could anaerobically degrade benzene coupled to nitrate reduction (Coates et al., 2001). At class level (**Figure 3D**), the SOB in the lower temperature reservoirs (LZ and QZ) belonged primarily to class *Alphaproteobacteria* (24.1 and 56.4%), whereas the SOB in the high temperature reservoirs (DQ and SL) belonged primarily to *Betaproteobacteria* (70.1 and 45.9%). *Epsilonproteobacteria* was the shared dominated class for LZ and SL reservoirs (35.2 and 32.8%).

As shown in **Figure 4B**, the SOB communities from the same reservoir were clustered more closely than those from other reservoirs. Furthermore, a phenomenon was observed that the groups of SOB in targeted reservoirs were reservoir specific. Expectedly, SOB communities were mostly affected by the same physicochemical factors as SRB in each reservoir, suggesting that SRB and SOB as well as the sulfate reduction and sulfur oxidation processes were closely inter-related and influenced each other. Temperature had a considerable influence on the SRB community in SL and DQ reservoirs compared with LZ and QZ blocks. In the relatively low temperature reservoirs (LZ and QZ), the pH, mineralization of formation brines and the concentration of sulfate ions were the main factors influencing the SOB population.

### The Abundance of SRB and SOB in Petroleum Reservoirs

**FIGURE 5:**
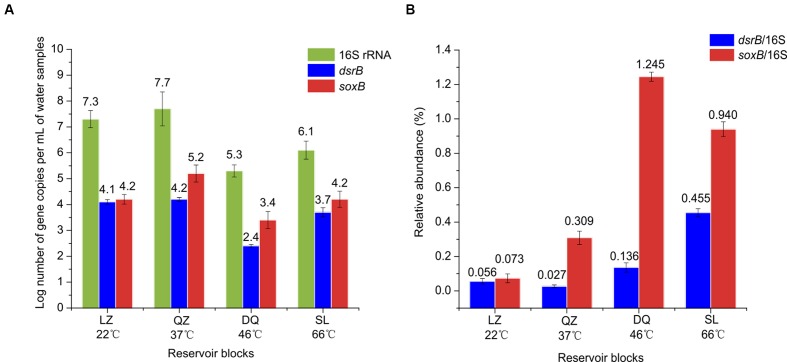
**Results of qPCR**. Abundance of copy numbers of 16S rRNA and *dsrB* and *soxB* genes **(A)**, and the ratios of *dsrB*/16S rRNA and *soxB*/16S rRNA **(B)**.

Quantitative real-time PCR was conducted to determine the abundance of SRB, SOB and total bacteria in petroleum reservoirs using *dsrB*, *soxB* and 16S rRNA genes (**Figure 5A**). The efficiency values for *dsrB*, *soxB* and 16S rRNA genes were 0.96, 1.03, and 1.02, respectively, with *R* > 0.99. The microorganisms showed significant difference in abundance across different reservoirs. The abundance of the 16S rRNA gene ranged from 2.05 × 10^5^ to 4.74 × 10^7^ copies/mL. The abundance of the *dsrB* and *soxB* genes ranged from 2.79 × 10^2^ to 1.26 × 10^4^ copies/mL and 2.55 × 10^3^ to 1.46 × 10^5^ copies/mL, respectively. The abundance of SRB in oil field samples ranged from 4.50 × 10^2^ to 4.50 × 10^7^ copies per reaction, based on the quantification of *dsrB* gene (Agrawal and Lal, 2009). In the present study, we used the ratios of *dsrB* and *soxB* to the 16S rRNA copy numbers in order to determine the relative abundances of SRB and SOB in the whole community. The relative abundances of SRB in the LZ, QZ, DQ, and SL reservoirs were 0.06, 0.03, 0.14, and 0.46%, respectively, while for SOB 0.07, 0.31, 1.25, and 0.94% were calculated, respectively (**Figure 5B**).

## Discussion

**FIGURE 6:**
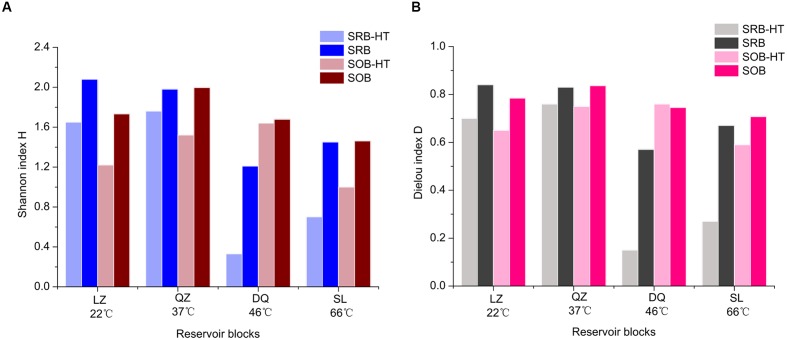
**Comparison of α-diversity indices of SRB and SOB retrieved from results of the high-throughput sequencing (reservoir-HT) and merged results (reservoir)** (**A**, Shannon H; **B**, Pielou E).

In this study, a comparative analysis of the sulfur cycle-related microbial communities in different petroleum reservoirs was performed using a combined approach with 16S rRNA gene high-throughput sequencing and functional gene clone libraries. The combination of these molecular techniques provided us with a detailed description of the SRB and SOB populations. Results were merged in the percentage level and higher Shannon indices and higher evenness were gained (**Figure 6**). Different means employed, e.g., 16S rRNA gene (physiological capacities of bacteria) and functional genes (specific metabolic pathways of bacteria) to identify bacteria, detected some common ones, including *Desulfotignum*, *Desulfomicrobium*, *Desulfotomaculum*, *Desulfomicrobium*, *Desulfovibrio*, *Paracoccus*, *Rhizobium*, *Thiobacillus*, *Hydrogenophaga*, *Dechloromonas*. Special genera were detected by 16S rRNA pyrosequencing and functional gene clone libraries. *Desulfitobacter*, *Desulfocurvus* and *Desulfomonile* were only detected by 16S rRNA gene sequencing. For potential SOB, bacteria of *Epsilonproteobacteria* were only detected by 16S rRNA sequencing and this was encountered by other studies due to the bias of primers used (Tourna et al., 2014). For *dsrB* and *soxB* genes clone libraries, uncultured taxa were detected indicating that the sulfur-related bacteria community has considerable potential for further exploration. Apart from uncultured taxa, bacteria that are not classified to SOB were detected by *soxB* gene clone libraries, like *Dechloromonas*. This indicated that *Dechloromonas* has the potential to oxidize the sulfur materials, despite they may get the *soxB* gene from other bacteria (lateral gene transfer).

Sulfate-reducing bacteria and SOB have been investigated in diverse ecological habitats; these bacteria are known to be of great significance in the carbon and sulfur cycles of the ecosystem and have a wide variety of applications, such as odor elimination, wastewater treatment, and remediation of pollutants (Belila et al., 2013; Varon-Lopez et al., 2013; Cao et al., 2014; Xia et al., 2014). Studying the distribution and composition of SRB and SOB can shed significant insights into the potential roles they play in ecological environments. From our results we found that diverse SRB and SOB members were widely distributed in petroleum reservoirs and SRB detected were strict anaerobic ones with a lower redox potential compared with most of the detected SOB. The detected SRB were affiliated to *Deltaproteobacteria* and *Clostridia*, while most microbes assigned to SOB were affiliated to *Beta-*, *Alpha-* and *Epsilonproteobacteria* which were found in many studies as is shown in **Figures 3C,D** (Belila et al., 2013; Guan et al., 2013; Headd and Engel, 2013; Gao et al., 2015a). Among petroleum reservoirs, SRB and SOB communities exerted niche specificity and showed strong relationships to reservoir physicochemical factors which play an important role in shift the microbial community, like temperature, PH and the concentration of various ions (Zhang et al., 2010, 2012; Xia et al., 2014; Xiao et al., 2016). Communities among different reservoirs were affected by different environmental variables, while SRB and SOB were influenced by same environmental variables in each reservoir.

**FIGURE 7:**
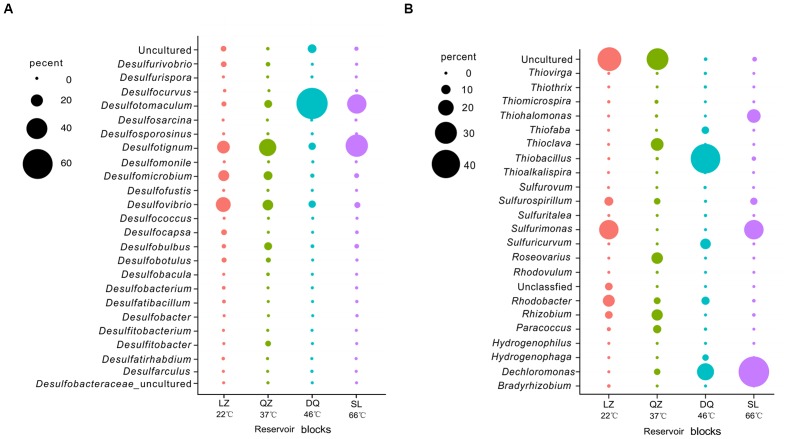
**The relative proportion of the SRB and SOB population in target reservoirs retrieved from merged results** (**A**, SRB; **B**, SOB).

Difference was more obvious in the community structure rather than diversity For SRB. This finding was consistent with that of Guan et al. (2013), they found that similar but structurally different sulphidogenic prokaryotes communities within the waters retrieved from corroding high temperature petroleum reservoirs. As shown in **Figure 7A**, certain dominant SRB populations were common in the three samples but with different proportions. For example, *Desulfotignum*, which was one of the shared dominant populations with its relative abundances in the different reservoirs ranging from 9.7 to 43.2%. Li et al. (2016) also found *Desulfotignum* is the most dominant SRP in high temperature and corrosive petroleum reservoirs. For SOB assembly, differences were observed in the diversity and compositions among petroleum reservoirs (**Figure 7B**). Apart from the community composition, differences were also observed in the abundance, which can affect the metabolic intensity. In our study, SRB and SOB were observed to have a higher relative abundance in the reservoirs with moderate to high temperatures, indicating that sulfur metabolism actively occurs in these reservoirs. This finding is in accordance with the opinion that the sulfur cycle may evolve in a high-temperature environment (Stetter et al., 1990). Voordouw et al. (2011) found that temperature was the controlling factor for sulfide production in reservoirs.

As we detected, SRB and SOB were widely inhabited in reservoirs with high diversity and abundance, what is the relationship between SRB and SOB? What role do SOB play in the reservoir souring and corrosion? Recently, An et al. (2016) found that sulfur-metabolizing *Epsilonproteobacteria* of the genera *Sulfuricurvum* and *Sulfurovum* are potentially powerful contributors to microbial corrosion in pipelines injected with bisulfite. Similar phenomenon was found by Okabe et al. (2007) that hydrogen sulfide is firstly produced by SRB under anaerobic conditions, and then SOB oxidize the dissolved H_2_S and other sulfur compounds to sulfuric acid, which corrodes the concrete in sewer pipes. Belonged to *Epsilonproteobacteria*, *Arcobacter*, and *Sulfurospirillum* have the metabolic capacity of sulfide oxidation with nitrate and were enriched by the nitrate-amendment rig which suffered serious corrosion compared with non-amendment rig (Drønen et al., 2014; Roalkvam et al., 2015). Also, the presence of sulfide-oxidizing and nitrate-reducing bacteria can promote the formation of greigite (Fe_3_S_4_), a product of corrosion (Drønen et al., 2014). In petroleum reservoirs, compared with hydrogen sulfide, more serious corrosion maybe brought about by sulfuric acid, and the SOB community should not be ignored. However, Nemati et al. (2001) have pointed out that some SOB could control SRB to produce biogenic sulfide possibly by increasing the environmental redox potential. Also, many SOB were detected and classified as NR-SOB and their activity was the primary force to control souring in nitrate injection system (Hubert and Voordouw, 2007). *Thiobacillus denitrificans* and *Sulfurimonas denitrificans*, commonly found in reservoirs, have the ability to couple denitrification to sulfur-compound oxidation (Beller et al., 2006; Sievert et al., 2008). But, no clear relationship was made by our present results since both SRB and SOB varied under different conditions. But no matter what is the relationship between SRB and SOB, study on the diversity, distribution of SRB and SOB community is essential to know and prevent biocorrosion.

Overall, the combined approach of 16S rRNA high-throughput sequencing and *dsrB* and *soxB* clone libraries represent an excellent approach for analyzing the diversity of SRB and SOB. Here, we found SRB and SOB were widely inhabited in oil reservoirs. Most detected SRB were affiliated to the *Deltaproteobacteria* and *Clostridia* classes, while most SOB belonged to *Alphaproteobacteria*, *Betaproteobacteria*, and *Epsilonproteobacteria*. The distribution of SRB and SOB showed high niche specificity and they were strong correlation to environmental variables such as temperature of the reservoir, pH of the formation brine, and sulfate concentration. But for the role of SOB in corrosion, no conclusion can be confirmed from our current data. In that, further studies are considered to focus on this issue. Together, these results improve our knowledge of the microbial populations involved in the sulfur cycle in reservoir environments.

## Author Contributions

HT, PG, GL, and TM conceived and proposed the idea. ZC, YansL, YanL, and JZ carried out the experiments and conducted data analysis. HT and TM drafted the manuscript. All authors have read and approved the final manuscript.

## Conflict of Interest Statement

The authors declare that the research was conducted in the absence of any commercial or financial relationships that could be construed as a potential conflict of interest.

## Footnotes

1 http://www.mothur.org

2 http://www.ncbi.nlm.nih.gov/tools/vecscreen/

3 http://greengenes.lbl.gov/cgi-bin/nph-index.cgi

4 http://www.ncbi.nlm.nih.gov/
