# Supplementary material for: Compositions and Abundances of Sulfate-Reducing and Sulfur-Oxidizing Microorganisms in Water-Flooded Petroleum Reservoirs with Different Temperatures in China
Source: Front Microbiol. 2017 Feb 2;8:143. doi: 10.3389/fmicb.2017.00143 (PMC5288354; doi:10.3389/fmicb.2017.00143)
Supplement: Supplementary file 1 [file Presentation_1.PDF]

Journal name:

Frontiers in Microbiology

Title:

Molecular detection of sulfate-reducing and sulfur-oxidizing microorganisms in water-flooding petroleum reservoirs

Authors:

Huimei Tian • Peike Gao • Zhaohui Chen • Yanshu Li • Yan Li • Yansen Wang • Jiefang Zhou • Guoqiang Li • Ting Ma*

Affiliations:

H. Tian • P. Gao • Z. Chen • Y. Li • Y. Li • Y. Wang • J. Zhou • G. Li • T. Ma*

Key Laboratory of Molecular Microbiology and Technology, Ministry of Education, College of Life Sciences, Nankai University, Tianjin 300071, P. R. China.

***Correspondence:**

Ting Ma

Mailing address: College of Life Sciences, Nankai University, Tianjin 300071, P. R. China

Tel/Fax: +86-22-23498185

E-mail: **tingma@nankai.edu.cn**

Supplementary Tables: Table S1 to S3

Table S1 Potential SRB genus inferred from 16S rRNA gene pyrosequencing and their affiliations and proportions in targeted reservoirs

Class	family	genus	Proportion of total SRB (%)			
			LZ	QZ	DQ	SL
Deltaproteobacteria	*Desulfobacteraceae*	*Desulfotignum*	8.47	37.79	0.00	85.56
Deltaproteobacteria	*Desulfobacteraceae*	*Desulfobotulus*	1.69	2.48	0.00	0.43
Deltaproteobacteria	*Desulfobacteraceae*	*Desulfatirhabdium*	1.69	0.57	0.00	0.00
Deltaproteobacteria	*Desulfobacteraceae*	*Desulfitobacterium*	0.00	0.19	0.00	0.00
Deltaproteobacteria	*Desulfobacteraceae*	*Desulfobacula*	0.00	2.29	0.00	0.00
Deltaproteobacteria	*Desulfobacteraceae*	*Desulfobacterium*	0.00	0.00	0.24	0.00
Deltaproteobacteria	*Desulfobacteraceae*	*Desulfosarcina*	0.00	0.00	0.00	0.06
Deltaproteobacteria	*Desulfobacteraceae*	*Desulfococcus*	0.00	0.00	0.00	0.24
Deltaproteobacteria	*Desulfobacteraceae*	*Desulfobacter*	0.00	0.00	0.00	1.04
Deltaproteobacteria	*Desulfovibrionaceae*	*Desulfovibrio*	50.85	5.34	6.35	2.08
Deltaproteobacteria	*Desulfomicrobiaceae*	*Desulfomicrobium*	5.08	25.95	0.71	0.61
Deltaproteobacteria	*Desulfobulbaceae*	*Desulfobulbus*	3.39	5.53	0.00	5.14
Deltaproteobacteria	*Desulfobulbaceae*	*Desulfocapsa*	11.86	0.00	0.00	2.45
Deltaproteobacteria	*Desulfobulbaceae*	*Desulfurivibrio*	11.86	6.68	0.00	0.61
Deltaproteobacteria	*Desulfobulbaceae*	*Desulfofustis*	0.00	0.19	0.00	0.06
Deltaproteobacteria	*Desulfarculaceae*	*Desulfarculus*	0.00	0.57	0.00	0.12
Deltaproteobacteria	*Syntrophaceae*	*Desulfomonile*	0.00	0.00	0.24	0.00
Clostridia	*Peptococcaceae*	*Desulfotomaculum*	0.00	0.00	92.24	0.00

Clostridia	*Peptococcaceae*	*Desulfosporosinus*	0.00	0.19	0.00	0.00
Clostridia	*Peptococcaceae*	*Desulfurispora*	0.00	0.00	0.24	0.00
Total proportion of bacteria (%)			0.98	0.58	0.77	13.57

Table S2 Potential SRB genus inferred from 16S rRNA gene pyrosequencing and their affiliations and proportions in targeted reservoirs

Class	family	genus	Proportion of total SOB (%)			
			LZ	QZ	DQ	SL
Alphaproteobacteria	*Rhodobacterales*	*Rhodovulum*	0.00	0.25	0.00	0.00
Alphaproteobacteria	*Rhodobacterales*	*Paracoccus*	0.00	0.46	0.00	0.00
Alphaproteobacteria	*Rhodobacterales*	*Rhodobacter*	0.00	2.83	15.26	0.00
Alphaproteobacteria	*Rhodobacterales*	*Roseovarius*	0.00	26.96	0.00	0.00
Alphaproteobacteria	*Rhodobacterales*	*Thioclava*	0.00	30.78	0.00	0.00
Betaproteobacteria	*Rhizobiales*	*Bradyrhizobium*	0.00	0.00	0.20	0.00
Betaproteobacteria	*Rhizobiales*	*Rhizobium*	14.81	26.08	0.20	0.05
Betaproteobacteria	*Burkholderiales*	*Hydrogenophaga*	0.00	0.05	6.85	0.00
Betaproteobacteria	*Hydrogenophilales*	*Thiobacillus*	0.00	0.00	0.00	0.27
Betaproteobacteria	*Rhodocyclales*	*Dechloromonas*	0.00	0.20	36.40	0.00
Betaproteobacteria	*Rhodocyclales*	*Hydrogenophilus*	0.00	0.00	0.20	0.00
Betaproteobacteria	*Rhodocyclales*	*Sulfuritalea*	0.00	0.00	0.78	0.00
Betaproteobacteria	*Rhodocyclales*	*Rhodocyclaceae_uncultured*	14.81	0.00	0.00	0.00
Gammaproteobacteria	*Chromatiales*	*Thiovirga*	0.00	0.00	0.78	0.00
Gammaproteobacteria	*Chromatiales*	*Thiofaba*	0.00	0.00	14.29	0.00
Gammaproteobacteria	*Chromatiales*	*Thioalkalispira*	0.00	0.22	0.00	0.00
Gammaproteobacteria	*Thiotrichales*	*Thiothrix*	0.00	0.00	0.39	0.00
Epsilonproteobacteria	*Campylobacterales*	*Sulfurospirillum*	18.52	11.17	0.59	13.36
Epsilonproteobacteria	*Campylobacterales*	*Sulfurimonas*	51.85	0.00	0.2	52.07

Epsilonproteobacteria	*Campylobacterales*	*Sulfurovum*	0.00	0.02	0.00	0.11
Epsilonproteobacteria	*Campylobacterales*	*Sulfuricurvum*	0.00	0.00	23.87	0.00
Total proportion of bacteria (%)			0.45	6.54	0.93	14.96

Table S3 Analysis of 16S rRNA sequences affiliated to SRB and SOB, and functional genes (*aprA*, *dsrB* and *soxB* genes) indices of high-throughput sequencing and clone libraries

Bacteria/ Gene	Reservoir	n.Genera/ OTUs	Shannon (H)	Simpson (D)	Dielou (E)	Coverage (%)
**(A)**						
SRB	LZ	10	1.65	0.70	0.72	
	QZ	14	1.76	0.76	0.67	
	DQ	6	0.33	0.15	0.18	
	SL	15	0.70	0.27	0.26	
SOB	LZ	4	1.22	0.65	0.88	
	QZ	12	1.52	0.75	0.61	
	DQ	13	1.64	0.76	0.64	
	SL	5	1.00	0.59	0.62	
**(B)**						
dsrB	LZ	28	1.83	0.80	0.83	92.4
	QZ	36	1.88	0.83	0.86	85.5
	DQ	38	2.00	0.82	0.81	88.0
	SL	24	1.02	0.45	0.49	91.0
soxB	LZ	24	1.37	0.67	0.66	91.7
	QZ	32	1.87	0.80	0.80	85.0
	DQ	19	0.75	0.30	0.36	83.0
	SL	36	0.73	0.30	0.35	90.0
